# Revisiting phylogenetic signal; strong or negligible impacts of polytomies and branch length information?

**DOI:** 10.1186/s12862-017-0898-y

**Published:** 2017-02-15

**Authors:** Rafael Molina-Venegas, Miguel Á. Rodríguez

**Affiliations:** Departamento de Ciencias de la Vida, Universidad de Alcalá, 28805 Alcalá de Henares, Madrid Spain

**Keywords:** BLADJ, Blomberg’s *K*, Pagel’s lambda, Phylogeny calibration, Phylogenetic resolution, Pseudo-chronograms

## Abstract

**Background:**

Inaccurate estimates of phylogenetic signal may mislead interpretations of many ecological and evolutionary processes, and hence understanding where potential sources of uncertainty may lay has become a priority for comparative studies. ﻿﻿﻿﻿Importantly, the sensitivity of phylogenetic signal indices and their associated statistical tests to incompletely resolved phylogenies and suboptimal branch-length information has been only partially investigated.﻿﻿﻿

**Methods:**

﻿﻿﻿Here, we use simulations of trait evolution along phylogenetic trees to assess whether incompletely resolved phylogenies (polytomic chronograms) and phylogenies with suboptimal branch-length information (pseudo-chronograms) could produce directional biases in significance tests (*p*-values) associated with Blomberg et al.’s *K* and Pagel’s lambda (λ) statistics, two of the most widely used indices to measure and test phylogenetic signal. Specifically, we conducted pairwise comparisons between the *p*-values resulted from the use of “true” chronograms and their degraded counterparts (i.e. polytomic chronograms and pseudo-chronograms), and computed the frequency with which the null hypothesis of no phylogenetic signal was accepted using “true” chronograms but rejected when using their degraded counterparts (type I bias) and vice versa (type II bias).

**Results:**

We found that the use of polytomic chronograms in combination with Blomberg et al.’s *K* resulted in both, clearly inflated estimates of phylogenetic signal and moderate levels of type I and II biases. More importantly, pseudo-chronograms led to high rates of type I biases. In contrast, Pagel’s λ was strongly robust to either incompletely resolved phylogenies and suboptimal branch-length information.

**Conclusions:**

Our results suggest that pseudo-chronograms can lead to strong overestimation of phylogenetic signal when using Blomberg et al.’s *K* (i.e. high rates of type I biases), while polytomies may be a minor concern given other sources of uncertainty. In contrast, Pagel’s λ seems strongly robust to either incompletely resolved phylogenies and suboptimal branch-length information. Hence, Pagel’s λ may be a more appropriate alternative over Blomberg et al.’s *K* to measure and test phylogenetic signal in most ecologically relevant traits when phylogenetic information is incomplete.

**Electronic supplementary material:**

The online version of this article (doi:10.1186/s12862-017-0898-y) contains supplementary material, which is available to authorized users.

## Background

Phylogenetic signal, i.e. the degree of phylogenetic constraint in species resemblance [1], is nowadays a central foundation for many disciplines in evolutionary ecology research, including macroecology [2, 3], macroevolution [4–6], conservation biology [7], and the recently emerged field of community phylogenetics [8, 9]. Importantly, inaccurate estimates of phylogenetic signal may mislead interpretations of many ecological and evolutionary processes [10, 11], and hence understanding where potential sources of uncertainty may lay has become a priority for comparative studies.

**Fig. 1:**
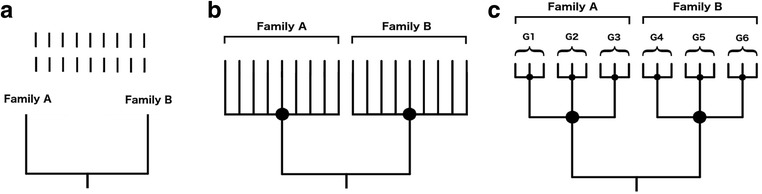
Schematic representation of the typical procedure to assembly supertrees by sticking missing species to a well-resolved backbone tree using taxonomy as a guide. Vertical bars represent missing species (**a**). Based on taxonomical knowledge, half of the species are classified in the families A and B, respectively, and thus they are added as relatively deep polytomies in the most derived node that unequivocally contains the species (**b**). Within each family, the species are classified in three different genera (G1-G6), and they are consequently regrouped as terminal polytomies following the same rationale as in the previous step (**c**)

The rapid increase of available molecular data, published phylogenies, and major advances in phylogenetic methods have allowed analyses involving phylogenies with dozens to hundreds of species (e.g. [12, 13]). Although the phylogenetic position of many species remains unresolved [14], deep phylogenetic relationships are relatively well-known for some lineages, thus constituting “backbone” working phylogenies for different groups of organisms such as flowering plants [15] and birds [16, 17]. An extended approach to build more complete phylogenies is to assemble supertrees combining these backbone phylogenies with smaller, overlapping trees [18, 19], and then add missing species as polytomies using taxonomy as a guide (e.g. [12]; see Fig. 1). However, the branching structure of the resulting supertrees, which usually have numerous terminal polytomies and few deeper polytomies, may lead to distorted estimates of phylogenetic signal [20].

**Fig. 2:**
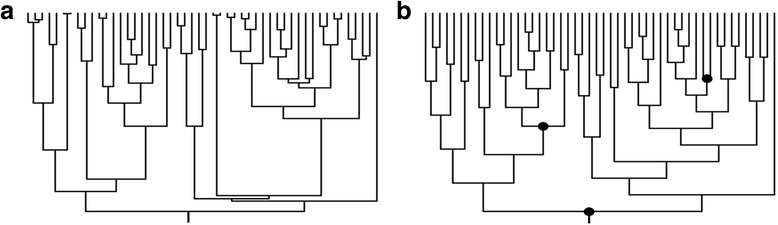
Comparison between the branching structure of a simulated “true” chronogram (**a**) and that of the same tree topology with branch lengths reassigned after using BLADJ (pseudo-chronogram) (**b**). The highlighted nodes in the pseudo-chronogram were fixed, and unknown nodes were placed evenly between fixed nodes. Note the marked difference in branch length variability between both branching structures

Another shortcoming of supertrees is that they usually lack accurate branch-length information. Because supertrees are constructed by assembling, grafting or subsetting published phylogenies from different sources, branch-length data is missing (i.e. the resultant supertrees only provide topological information) and it has to be added afterwards [21]. For example, many plant community ecology studies conducted over the last decade have made use of phylogenetic hypotheses derived from supertree topologies (e.g. APG IV [15]) calibrated with the Branch Length Adjuster algorithm (BLADJ). This algorithm (implemented in Phylocom software [22]) assigns published age divergences (provided by the user) to particular nodes in the target topology, and then places the remaining nodes evenly between them. The resulting time-calibrated trees are actually pseudo-chronograms that show lower ﻿variability in ﻿branch length than well-calibrated phylogenies –i.e. using molecular clocks (divergence-time estimates based on nucleotide substitutions per site; [23], Fig. 2). Although the use of pseudo-chronograms has become common practice in some fields of evolutionary ecology such us community phylogenetics, the extent to which pseudo-branch lengths could affect estimates of phylogenetic signal has been only partially addressed (see [24], and below).

Amongst the indices that quantify phylogenetic signal in continuous traits (see [24] for an extensive review), Blomberg et al.’s *K* [1] and Pagel’s lambda (λ) [25] are the most widely used in ecology. Both indices assume the classic Brownian motion (BM) evolutionary model (i.e. random walk divergence in species resemblance), and their values vary from 0 to 1 for λ and from 0 to > > 1 for *K*. In both cases, values close to 0 indicate no phylogenetic signal –the trait has evolved independently of phylogeny and close relatives are not more similar than distant relatives–; values close to 1 indicate trait evolution according to BM; and, in the case of *K*, values >1 reflect that close relatives are more similar than expected under BM. Unlike other phylogenetic signal metrics, being model-based provides both indices with the advantage of allowing direct comparison of phylogenetic signal strengths not only across traits but also across different phylogenetic trees. This is at least known to be the case for well resolved phylogenies with accurate branch lengths. But how reliable would *K* and λ be when applied to low-quality phylogenetic trees?

This question has been partially addressed by Münkemüller et al. [24] by comparing simulated phylogenies with and without terminal polytomies (i.e. polytomies that occur towards the phylogenetic tips). These authors concluded that both indices and their associated statistical tests were virtually unaffected by terminal polytomies. Still, they could not discard that polytomies occurring deeper in the phylogeny –as in real supertrees (e.g. [26]; see Fig. 1) – could lead to biased estimates. Interestingly, in a similar simulation analysis focussed on Blomberg et al.’s *K* Davies et al. [20] found precisely this; i.e. that *K* yielded inflated estimates of phylogenetic signal in highly polytomic trees at both terminal and deeper levels. However, these authors did not investigate the effects of polytomies on either the statistical significance of *K* or the performance of λ.

Münkemüller et al. [24] also explored the effects of omitting branch length information (i.e. setting all branches to unity) and only found that Blomberg et al.’s *K* statistical test responded slightly positively to this treatment (i.e. lower, more statistically significant *p*-values than those obtained when using “true” branch lengths). Given other potential sources of uncertainty, they interpreted this as a negligible effect. However, their conclusion contrasts with propositions advanced by Pavoine & Ricotta [27], who, based on the underlying mathematics of *K*, hypothesized that lacking branch lengths could decrease the power of this index to detect phylogenetic signal. Thus, the extent to which the quality of available branch length information could affect this index remains unclear.

Here, we use simulations of trait evolution along phylogenetic trees to assess whether incompletely resolved phylogenies (polytomic chronograms) and phylogenies with suboptimal branch length information (pseudo-chronograms calibrated with BLADJ) could produce directional biases in significance tests associated with Blomberg et al.’s *K* and Pagel’s λ.

## Methods

### Phylogenetic trees simulations

We used the function *pbtree* in phytools R package [28] to obtain simulated, fully-resolved and perfectly dated phylogenies (hereafter “true” chronograms). Specifically, we generated five sets of pure-birth ultrametric phylogenies (*N* = 1000 phylogenies per set) containing *n* species (tips), with *n* equal to 50, 100, 200, 400 and 1000 respectively (see [24] for a similar approach). This dataset comprises a wide array of phylogenies of varying degrees of tree stemminess and tree imbalance (see Additional file 1: Figure S1), which prevent us from obtaining biased results due to tree shape. Nevertheless, we also tested for potential effects of tree shape on the results (see below).

We derived two types of distorted phylogenies from the “true” chronograms. The first type was intended to replicate common patterns of polytomy distribution in commonly-used supertrees, which usually show a high density of terminal polytomies and few deeper polytomies (e.g. supertrees based on the backbone topology provided by APG IV for angiosperm plants [15]). To do so, we followed two different node-collapsing strategies. First, we generated gradually unresolved phylogenies (hereafter polytomic chronograms) by randomly collapsing 20, 40, 60 and 80% of the nodes placed above half of the height of the “true” chronograms (shallow-nodes strategy). Second, we generated gradually unresolved phylogenies by randomly collapsing 20, 40, 60 and 80% of all the nodes of the “true” chronograms (all-nodes strategy). Note that although the latter strategy may lead to less realistic topologies than the former (i.e. high density of polytomies towards the root of the trees), it has been previously used to analyze robustness of Blomberg et al.’s *K* to incompletely resolved phylogenies [20].

**Fig. 3:**
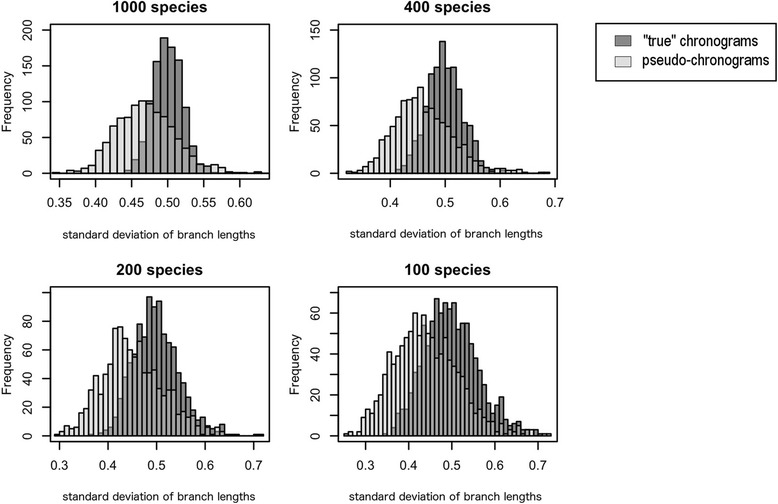
Frequency histograms showing differences in branch length variability (standard deviation) between “true” chronograms and pseudo-chronograms calibrated with BLADJ (fixing 5% of the nodes). Note that overall, pseudo-chronograms show lower branch length variability (i.e. branch lengths are more homogeneous) than perfectly dated “true” chronograms

The second type of distorted phylogenies consisted of pseudo-chronograms calibrated with BLADJ using a certain fraction of the whole set of node ages of the “true” chronograms (i.e. 5, 15, 25 and 35% of the nodes, respectively). To do so, we divided each “true” chronogram into five equally sized time-slices, and then selected a proportional number of nodes ﻿from﻿ each time-slice at random (with a minimum of one single node per time-slice), in such a way that the sum of selected nodes across time-slices was equal to the total number of nodes to be fixed for each treatment (i.e. 5, 15, 25 and 35% of the whole set of node ages of each “true” chronogram, respectively). The root-node was fixed in all cases, in order to retain the height of the “true” chronograms in the derived pseudo-chronograms. Subsequently, the ages of all non-fixed nodes were deleted, and replaced with pseudo-ages using BLADJ. That is, non-fixed nodes were assigned with new ages that distributed them evenly among the fixed nodes. This procedure was intended to replicate the branch length structure of commonly used pseudo-chronograms (typically in community phylogenetic studies), which show low variability in branch length compared to that of “true” chronograms (Figs. 2 and 3). Finally, in order to explore the potential interaction between polytomies and suboptimal branch-length information, we derived an extra set of polytomic pseudo-chronograms by applying the shallow node-collapsing strategy to the pseudo-chronograms as described above.

### Trait evolution with variable degree of phylogenetic signal

We simulated the evolution of continuous traits with varying degrees of phylogenetic signal using the *fastBM* function in phytools R package [28]. To do so, we first rescaled the “true” chronograms by multiplying the off-diagonal elements of the variance-covariance matrix by a down-weighting coefficient λ (ranging from 0.1 to 0.9), and then we simulated trait evolution along the branches of the rescaled phylogenies following a Brownian motion (BM) model of evolution (root value *a* = 0 and instantaneous variance σ^2^ = 1). Briefly, a BM model on a phylogeny describes purely neutral (random) evolution of a trait with variance proportional to the square root of branch lengths [29]. Thus, nine traits with varying degrees of phylogenetic signal were generated for each of the “true” chronograms. This procedure was intended to replicate the multiple scenarios of trait evolution leading to a continuum between very weak phylogenetic signal (close to random distribution of trait values across species, λ = 0.1) and some resemblance among close relatives (close to BM expectation, λ = 0.9).

### Directional biases in estimates of phylogenetic signal

We used the *phylosig* function in phytools R package [28] to obtain the values of Blomberg et al.’s *K* and Pagel’s λ statistics and their corresponding *p*-values for each simulated trait and “true” chronogram and its derived polytomic chronogram and pseudo-chronogram. The statistical significance of *K* was assessed based on comparison of the observed phylogenetically independent contrasts and the expected contrast under 999 randomizations [1], whereas the statistical significance of λ was assessed based on comparison of the likelihood a model accounting for the observed λ with the likelihood of a model that assumes complete phylogenetic independence [25] (both statistical tests are completely implemented within the *phylosig* function [28]).

**Fig. 4:**
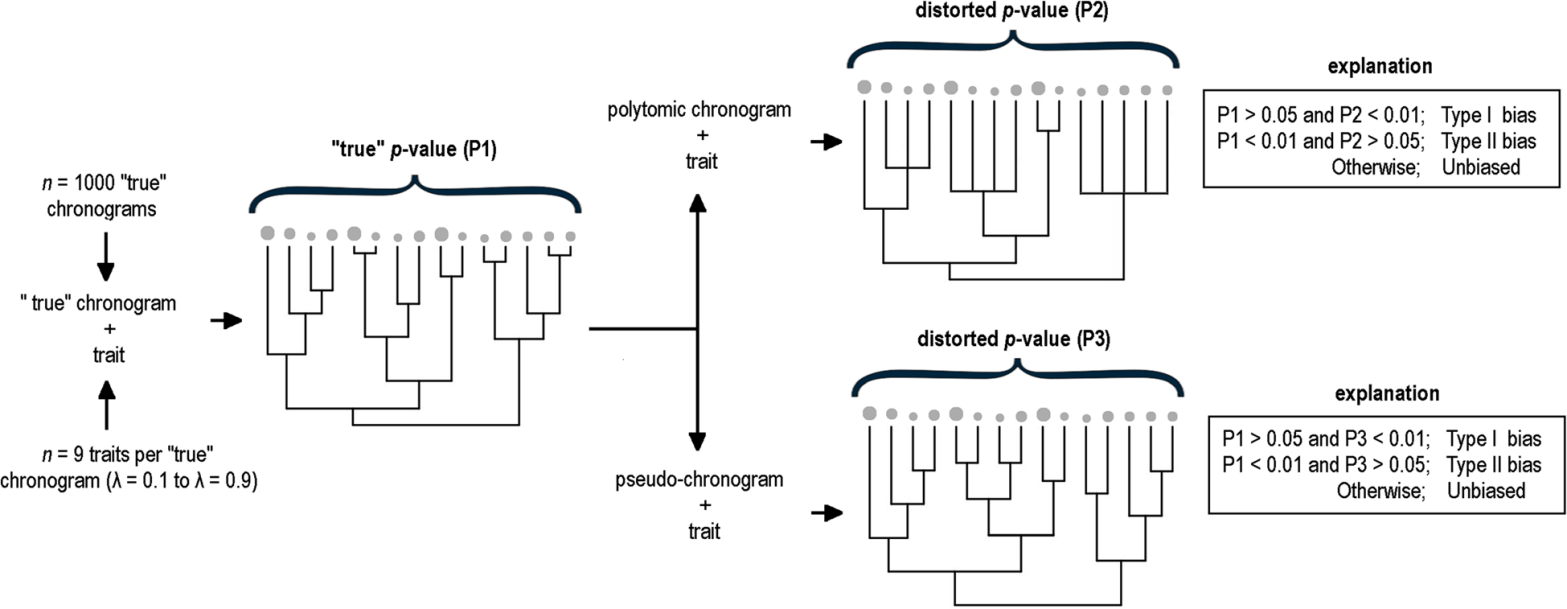
Schematic representation of the analysis conducted to quantify the frequency of strong shifts in the *p*-values (i.e. directional biases) derived from the use of polytomic chronograms (P2) and pseudo-chronograms (P3) instead of the “true” chronograms (P1)

We quantified the frequency of strong shifts in the *p*-values that occurred due to the use of polytomic chronograms and pseudo-chronograms instead of the “true” chronograms (Fig. 4). To do so, we focused on individual pairwise comparisons, each involving a “true” chronogram and its degraded counterpart. Specifically, we computed the frequency with which the null hypothesis of no phylogenetic signal was accepted using a “true” chronogram (nominal α = 5% level), but rejected when using its polytomic chronogram and pseudo-chronogram versions, respectively (nominal α = 1% level; type I biases). In addition, we used a similar procedure to quantify the extent to which both types of degraded chronograms led to type II biases. That is, we computed the frequency with which the null hypothesis of no phylogenetic signal was rejected using a “true” chronogram (nominal α = 1% level), but accepted using its polytomic chronogram and pseudo-chronogram versions, respectively (nominal α = 5% level). In both cases, we employed different nominal α-errors to screen out potential errors arising from marginally significant (or non-significant) *p*-values.

In order to test for potential effects of tree steaminess (i.e. the distribution of branching events within a tree [30]) on the results, we repeated the analyses described above considering only those trees that were below and above the 10 and 90 deciles of the distribution of the gamma statistic [30] within each sample size category, respectively. Low and high values of the gamma statistic correspond to phylogenetic trees that show longer inter-nodal distances towards the tips (“tippy” trees) and the root (“stemmy” trees), respectively (see Additional file 1: Figure S1). Similarly, in order to test for potential effects of tree imbalance on the results, we repeated the analyses described above considering only those trees that were below and above the 10 and 90 deciles of the distribution of the Colless’ statistic [31]. Low and high values of the Colless’ statistic correspond to phylogenetic trees that are highly balanced and unbalanced, respectively. All the analyses were conducted in R version 3.2.2 [32].

## Results

**Fig. 5:**
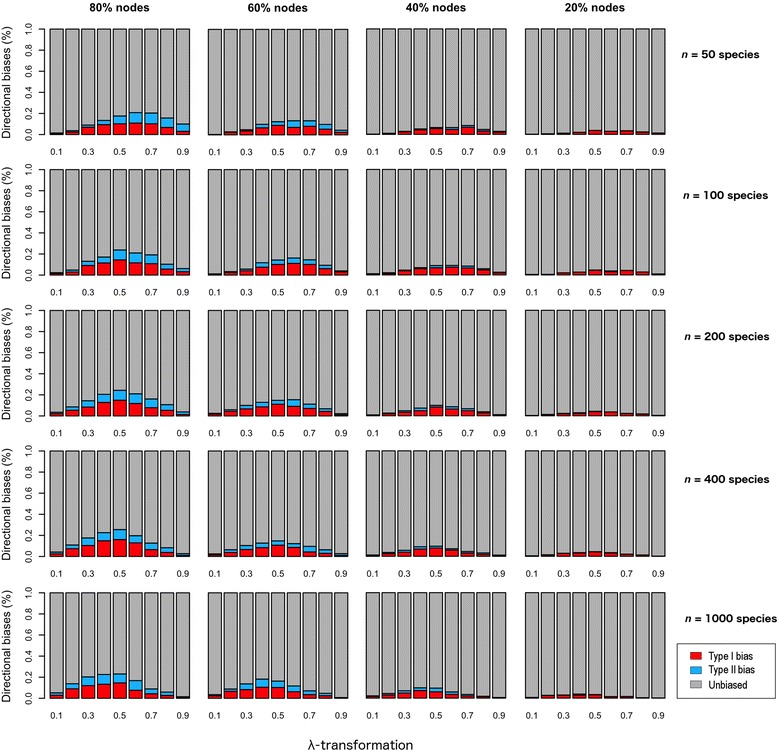
Graphical representation of the frequency of type I and II biases when quantifying phylogenetic signal using Blomberg et al.’s *K* and polytomic chronograms (shallow-nodes strategy). The x-axis represents the degree of phylogenetic signal in the traits (λ-transformations). The percentages above the figures refer to the nodes that were randomly collapsed to generate the polytomic chronograms (see Additional file 1: Figure S2 for results derived from an alternative node-collapsing strategy)

As expected, polytomic chronograms led to inflated estimates of phylogenetic signal using Blomberg et al.’s *K* (Additional file 2: Figure S1), but only resulted in moderate type I and II biases (Fig. 5). Both types of biases were more frequent at intermediate-to-high degrees of phylogenetic signal in small-sized phylogenies, and they shifted progressively towards intermediate-to-low degrees as phylogeny size increased. We found no significant differences between both node-collapsing strategies, which led to virtually identical results (see Additional file 1: Figure S2).

**Fig. 6:**
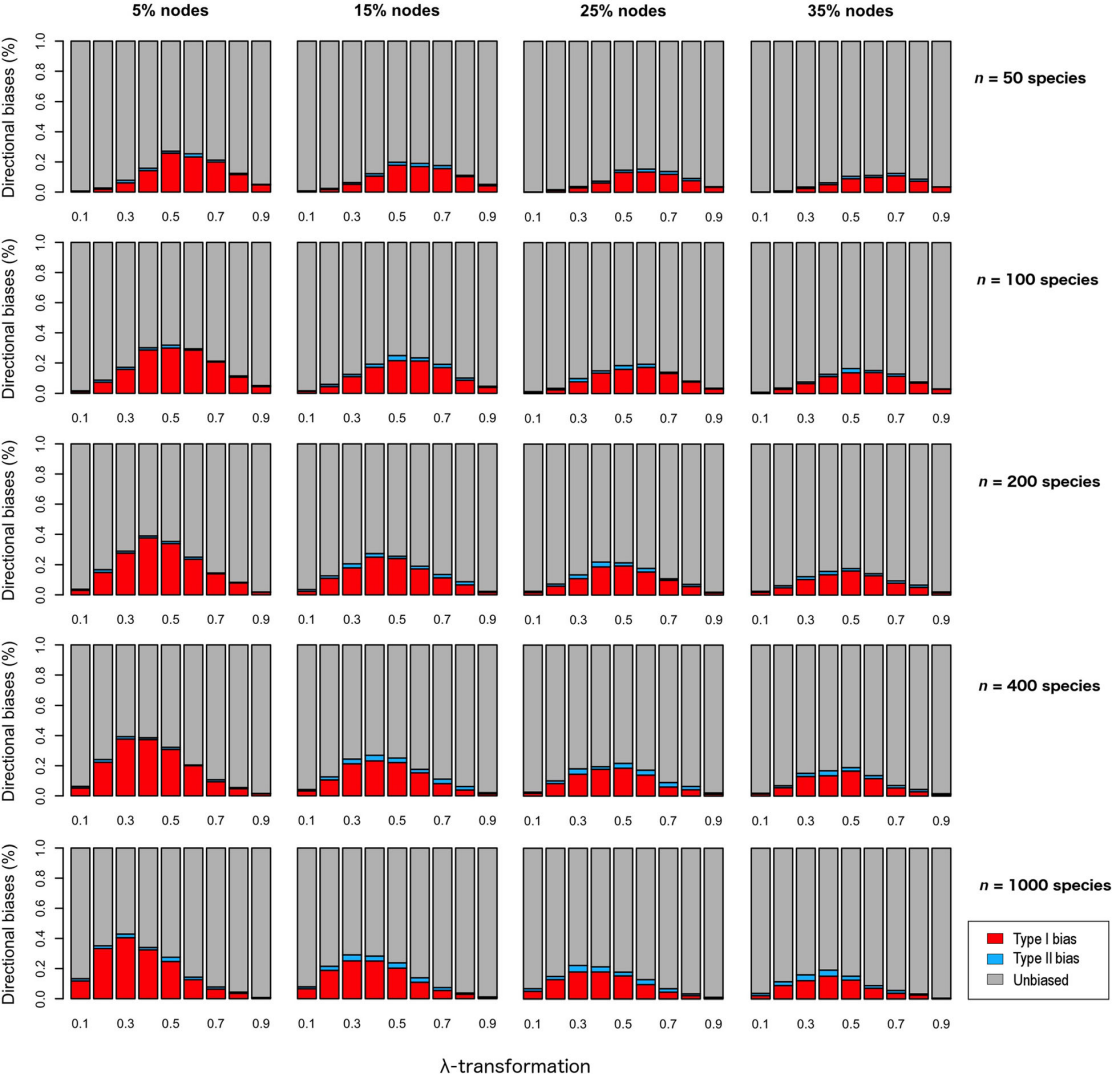
Graphical representation of the frequency of type I and II biases when quantifying phylogenetic signal using Blomberg et al.’s *K* and pseudo-chronograms calibrated with BLADJ. The x-axis represents the degree of phylogenetic signal in the traits (λ-transformations). The percentages above the figures refer to the nodes that were fixed to generate the pseudo-chronograms

The relatively poor performance of *K* caused by polytomies seemed less of a problem compared with the effect of pseudo-branch lengths. In this case, estimates of phylogenetic signal were also inflated (Additional file 2: Figure S2), and very high type I biases dominated at all instances (Fig. 6). Further, type I biases increased slightly with sample size. As well, the incidence of both types of bias shifted progressively from higher to lower degrees of phylogenetic signal as sample size increased. Overall, tree shape (i.e. tree stemminess and tree imbalance) was not a significant factor driving the observed directional biases (Additional file 1: Figure S3–S6). However, small-sized (*n* = 50 sp) balanced trees showed slightly higher type I biases due to pseudo-branch lengths than unbalanced trees (Additional file 1: Figure S6). Finally, we found no evidence for interaction between polytomies and pseudo-branch lengths on estimates of phylogenetic signal (Additional file 1: Figure S7).

Importantly, estimates of phylogenetic signal using Pagel’s λ were largely unaffected by polytomies and pseudo-branch lengths (Additional file 2: Figure S3 and S4), and both types of distorted chronograms showed type I and II biases below 5% in almost all cases (data not shown). Only small (*n*=50 sp), heavily polytomic trees (80% of nodes collapsed) showed slight levels of type II biases (between 5 and 10%).

## Discussion

Erroneous estimates of phylogenetic signal might mislead inferences drawn from evolutionary ecology studies and many downstream disciplines such us community phylogenetics, macroevolution and conservation biology. In this study, we focused on two of the most widely used indices to measure and test phylogenetic signal in ecological traits, and illustrated how polytomic chronograms and especially pseudo-chronograms calibrated with BLADJ, which have been extensively used in the literature (typically in the field of community phylogenetics), may frequently lead to spurious estimates of phylogenetic signal.

Previous work noticed that polytomies could produce directional biases in different phylogenetic analyses [33–35] and, importantly, Davies et al. [20] found that Blomberg et al.’s *K* yielded inflated phylogenetic signal estimates in highly polytomic trees. However, these authors did not check for the existence of directional biases in significance tests associated with *K*. We have done so here and only found moderate rates of type I and II biases, which might be of minor concern given other sources of uncertainty (i.e. suboptimal branch-length information). Further, although the optimal solution would be to invest in the necessary resources for producing fully-resolved phylogenies, directional biases associated to polytomies may be partially mitigated by applying either rarefaction-based solutions (e.g. [20, 36]) or model-based approaches [37]. Nonetheless, and despite the topology of many species-rich clades remains largely unresolved [14], it is theoretically a matter of time and effort before we get to make comprehensive, fully-resolved topologies.

However, our results suggest that non accurate branch lengths could be a much more pervasive problem than phylogenetic resolution. Previous work already pointed out the importance of branch length information in phylogenetic analyses (e.g. [38, 39]). Here, we have reported strong type I biases in estimates of phylogenetic signal using Blomberg et al.’s *K* and phylogenies with pseudo-branch lengths. This contrasts with Münkemüller et al.’s conclusion that the effect of branch length information is rather negligible for *K* (and other phylogenetic signal indices), despite these authors detecting lower *p*-values in the Blomberg et al.’s *K* tests derived from phylogenies missing branch lengths (i.e. significant but erroneous estimates of phylogenetic signal). We think the apparent differences between Münkemüller et al.’s results and ours arise simply from the way in which the data were analysed in each study. Unlike the individual pairwise comparisons we used here, Münkemüller et al. sought for significant differences between distributions of *p*-values as a whole, using general additive models (see “model-based sensitivity analyses” in [24]). Although this approach might be appropriated to elucidate strong directional trends in data, individual responses between particular “true” phylogenies and the corresponding degraded trees could have gone unnoticed, thus leading to underestimation of the effect of branch length information. Further, Pavoine & Ricotta [27] hypothesized that non-accurate or non-available branch lengths could decrease the power of Blomberg et al.’s *K* to detect phylogenetic signal, and warned against the use of this index when branch lengths are missing. Our results suggest that rather than decrease the power of the statistic, pseudo-branch lengths could lead to strong overestimation of the signal (i.e. high rates of type I biases).

Unlike Blomberg et al.’s *K* statistic, our results suggest that Pagel’s λ is strongly robust to either polytomies and pseudo-branch lengths. This is much in line with previous evidence that showed that Pagel’s λ is robust to incomplete phylogenetic information (i.e. omission of branch lengths) in phylogenetic comparative analyses [40]. However, Pagel’s λ has a clear disadvantage over Blomberg et al.’s *K*; the former will fail to detect phylogenetic signals stronger than Brownian motion expectation, as may occur in highly conserved traits (e.g. [41]). Nevertheless, it may be a minor concern regarding most ecologically relevant traits, which often exhibit phylogenetic signal below this threshold (i.e. *K* and λ < 1).

It is important to note that many studies that have made use of pseudo-chronograms calibrated with BLADJ to estimate phylogenetic signal using Blomberg et al.’s *K* do not specify the percentage of nodes that were fixed for branch length calibration (e.g. [42–45]), and it is often rather low, which may increase the risk to obtain spurious estimates of phylogenetic signal. For instance, in plant ecological studies, a fairly standardized practice for generating pseudo-chronograms with BLADJ is to use plant clade age estimates from Wikström et al. [46]. However, this set of calibration points (available in Phylocom package) includes only 120 clades at the family level or less than 30% of the 413 families recognized by APG IV [47]. Thus, given the strong sensitivity of Blomberg et al.’s *K* statistic to non-accurate branch lengths, estimates of phylogenetic signal that rely upon ﻿﻿﻿this index ﻿and ﻿pseudo-chronograms calibrated with BLADJ should be accompanied by detailed information about the calibration process (i.e. the number of nodes of the phylogeny that are fixed). As well, low but significant phylogenetic signals estimated with Blomberg et al.’s *K* on large-sized pseudo-chronograms should be interpreted with particular caution, given the probability of ﻿making type I biases﻿ when phylogenetic signal is rather low seems to increase with sample size.

The most notable feature of pseudo-chronograms calibrated with BLADJ is they show lower branch length variabilitythan well-calibrated trees (i.e. “true” chronograms; Fig. 3). Thus, our conclusions may also apply to other calibration methods that also generate pseudo-chronograms of artificially low variability in branch length (e.g. Graphen’s rho transformation [48]) in comparison with that expected from the true chronograms. It is worthy to mention that the branching pattern of the pure-birth trees used in our analyses may differ to some extent from that of real chronograms, which may limit the scope of the conclusions of the present study. Nevertheless, variability in branch length of real chronograms is expected to be higher than that of pseudo-chronograms, given the complex evolutionary dynamics that characterize natural evolution.

Finally, the distorted effects of polytomies and pseudo-branch lengths in estimates of phylogenetic signal could also affect other indices that show similar properties as Blomberg et al.’s *K*. For example, the phylogenetic signal-representation curve approach (PSR), a method for estimating phylogenetic signal built upon sequential phylogenetic eigenvector regression (PVR), has been demonstrated to strongly correlate with Blomberg et al.’s *K* [49]. Hence, the use of pseudo-chronograms in studies of phylogenetic signal and other phylogenetic analyses should be done with caution. Nevertheless and in the light of our results, Pagel’s λ seems a more appropriate alternative over Blomberg et al.’s *K* to measure and test phylogenetic signal in most ecologically relevant traits when phylogenetic information is incomplete.

## Conclusions

Our results suggest that pseudo-chronograms calibrated with BLADJ can lead to strong overestimation of phylogenetic signal when using Blomberg et al.’s *K* (i.e. high rates of type I biases), while polytomies may be a minor concern given other sources of uncertainty (i.e. incorrect branch lengths). Importantly, other calibration methods that also generate pseudo-chronograms of artificially low variability in branch length (e.g. Graphen’s rho transformation) may lead to similar spurious estimates of phylogenetic signal. In contrast, Pagel’s λ seems strongly robust to either polytomies and pseudo-branch lengths, and hence may be a more appropriate alternative over Blomberg et al.’s *K* to measure and test phylogenetic signal in most ecologically relevant traits when phylogenetic information is incomplete.

## Additional files

Additional file 1:
**Appendix 1.** (extra analyses). (ZIP 4769 kb)
Additional file 2:
**Appendix 2.** (values obtained for Blomberg et al.’s *K* and Pagels’s λ). (ZIP 4841 kb)
Additional file 3
**Appendix 3.** (R code to simulate “true” chronograms and to generate polytomic chronograms and pseudo-chronograms). (PDF 62 kb)
