# Erratum to: Revisiting phylogenetic signal; strong or negligible impacts of polytomies and branch length information?

**DOI:** 10.1186/s12862-017-0946-7

**Published:** 2017-05-18

**Authors:** Rafael Molina-Venegas, Miguel Á. Rodríguez

**Affiliations:** Departamento de Ciencias de la Vida, Universidad de Alcalá, 28805 Alcalá de Henares, Madrid Spain

## Erratum

The original version of this manuscript [1] contained a mistake regarding the definition of Brownian motion model of evolution. This mistake does not affect the results of the manuscript, but it should be clarified.

Page 4. Second paragraph reads:

“Briefly, a BM model on a phylogeny describes purely neutral (random) evolution of a trait with variance proportional to the square root of branch lengths”.

and it should read as follows:

“Briefly, a BM model on a phylogeny describes purely neutral (random) evolution of a trait with variance directly proportional to the branch lengths”.

Also, there are two typos in the text:

“steaminess” should be “steminess” (third paragraph in page 5)

“Graphen” should be “Grafen” (fifth paragraph in page 8 and Conclusions)

## Acknowledgements

We thank Dr. Simon Blomberg for pointing out the mistake.
