# Supplementary material for: Revisiting phylogenetic signal; strong or negligible impacts of polytomies and branch length information?
Source: BMC Evol Biol. 2017 Feb 15;17:53. doi: 10.1186/s12862-017-0898-y (PMC5312541; doi:10.1186/s12862-017-0898-y)
Supplement: Additional file 1: — Appendix 1. (extra analyses). (ZIP 4769 kb) [file 12862_2017_898_MOESM1_ESM.zip › Appendix 1 Figure S7.pdf]

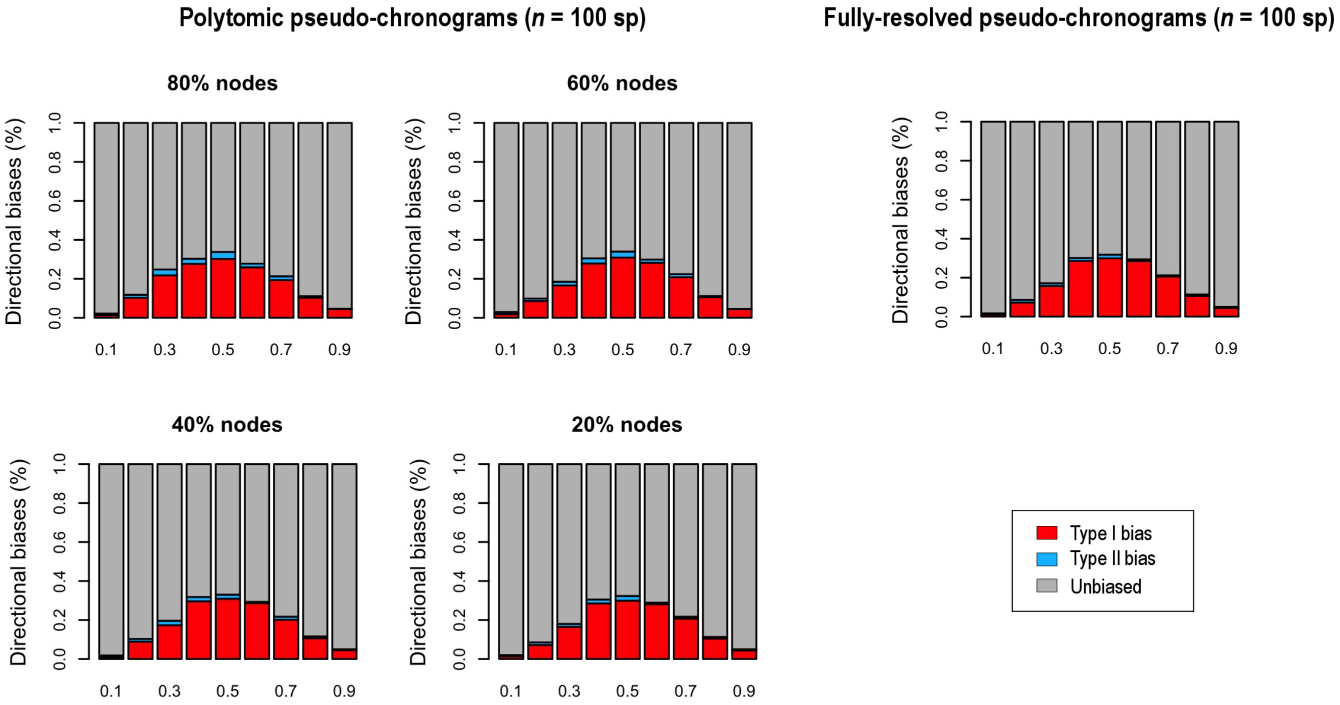

Figure S4. Graphical representation of the frequency of type I and II biases when quantifying phylogenetic signal using Blomberg et al.'s *K* and polytomic (shallow-nodes strategy) pseudo-chronograms calibrated with 5% of the nodes (n = 100 sp). The x-axis represents the degree of phylogenetic signal in the traits (λ-transformations). The percentages above the figures refer to the nodes that were randomly collapsed to derive polytomic pseudo-chronograms from the corresponding fully-resolved pseudo-chronograms (see main text). The plot on the right side of the figure shows the frequency of type I and II biases due to fully-resolved pseudo-chronograms (see Fig. 6). Note that the rate of statistical biases due to polytomic pseudo-chronograms remains similar to that of fully-resolved pseudo-chronograms irrespective of the degree of phylogenetic resolution.
