# Supplementary material for: Revisiting phylogenetic signal; strong or negligible impacts of polytomies and branch length information?
Source: BMC Evol Biol. 2017 Feb 15;17:53. doi: 10.1186/s12862-017-0898-y (PMC5312541; doi:10.1186/s12862-017-0898-y)
Supplement: Additional file 1: — Appendix 1. (extra analyses). (ZIP 4769 kb) [file 12862_2017_898_MOESM1_ESM.zip › Appendix 1 Figure S3.pdf]

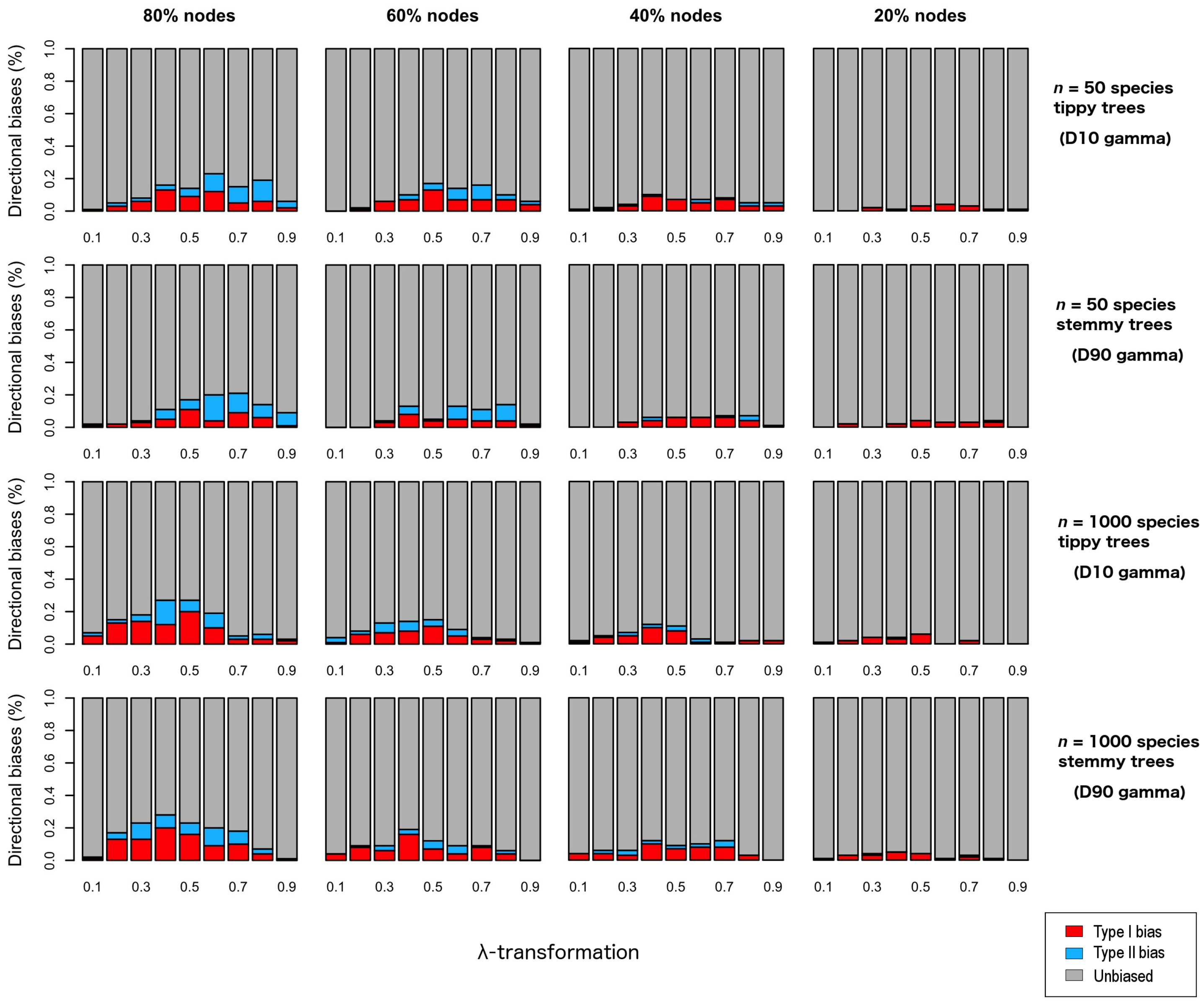

Figure S3. Graphical representation of the frequency of type I and II biases when quantifying phylogenetic signal using Blomberg et al.'s *K* and polytomic chronograms (shallow-nodes strategy) derived from extremelly tippy and stemmy "true" chronograms, respectively (i.e. trees below and above the 10 and 90 deciles of the distribution of the gamma statistic). The x-axis represents the degree of phylogenetic signal in the traits (λ-transformations). The percentages above the figures refer to the nodes that were randomly collapsed to generate the polytomic chronograms (see main text). Only results for n = 50 and n = 1000 sp are shown.
