# Supplementary material for: Revisiting phylogenetic signal; strong or negligible impacts of polytomies and branch length information?
Source: BMC Evol Biol. 2017 Feb 15;17:53. doi: 10.1186/s12862-017-0898-y (PMC5312541; doi:10.1186/s12862-017-0898-y)
Supplement: Additional file 1: — Appendix 1. (extra analyses). (ZIP 4769 kb) [file 12862_2017_898_MOESM1_ESM.zip › Appendix 1 Figure S1.pdf]

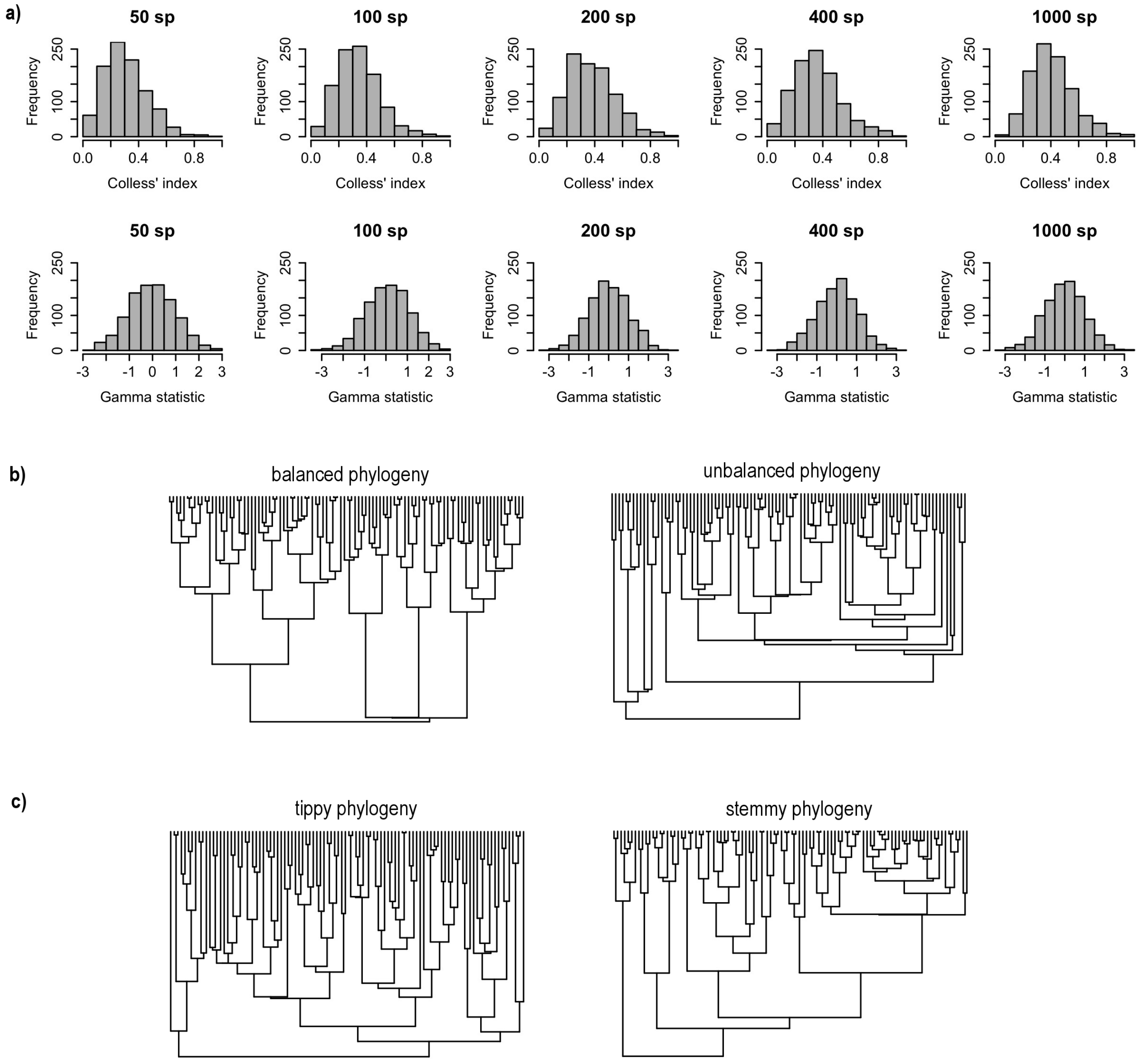

Figure S1. (a) Frequency histograms showing the distribution of values of the Colless' index (tree imbalance, top pannels) and the gamma statistic (tree stemminess, bottom pannels) calculated for the "true" chronograms used in the study. The values of the Colless' index were standardized between 0 and 1 within each size category. (b) The two chronograms of the dataset (n = 100 sp) that showed minimum (left) and maximum (right) values of the Colless' index, respectively. (c) The two chronograms of the dataset (n = 100 sp) that showed minimum (left) and maximum (right) values of the gamma statistic, respectively.
