# Supplementary material for: Revisiting phylogenetic signal; strong or negligible impacts of polytomies and branch length information?
Source: BMC Evol Biol. 2017 Feb 15;17:53. doi: 10.1186/s12862-017-0898-y (PMC5312541; doi:10.1186/s12862-017-0898-y)
Supplement: Additional file 2: — Appendix 2. (values obtained for Blomberg et al.’s K and Pagels’s λ). (ZIP 4841 kb) [file 12862_2017_898_MOESM2_ESM.zip › Appendix 2 Figure S2.pdf]

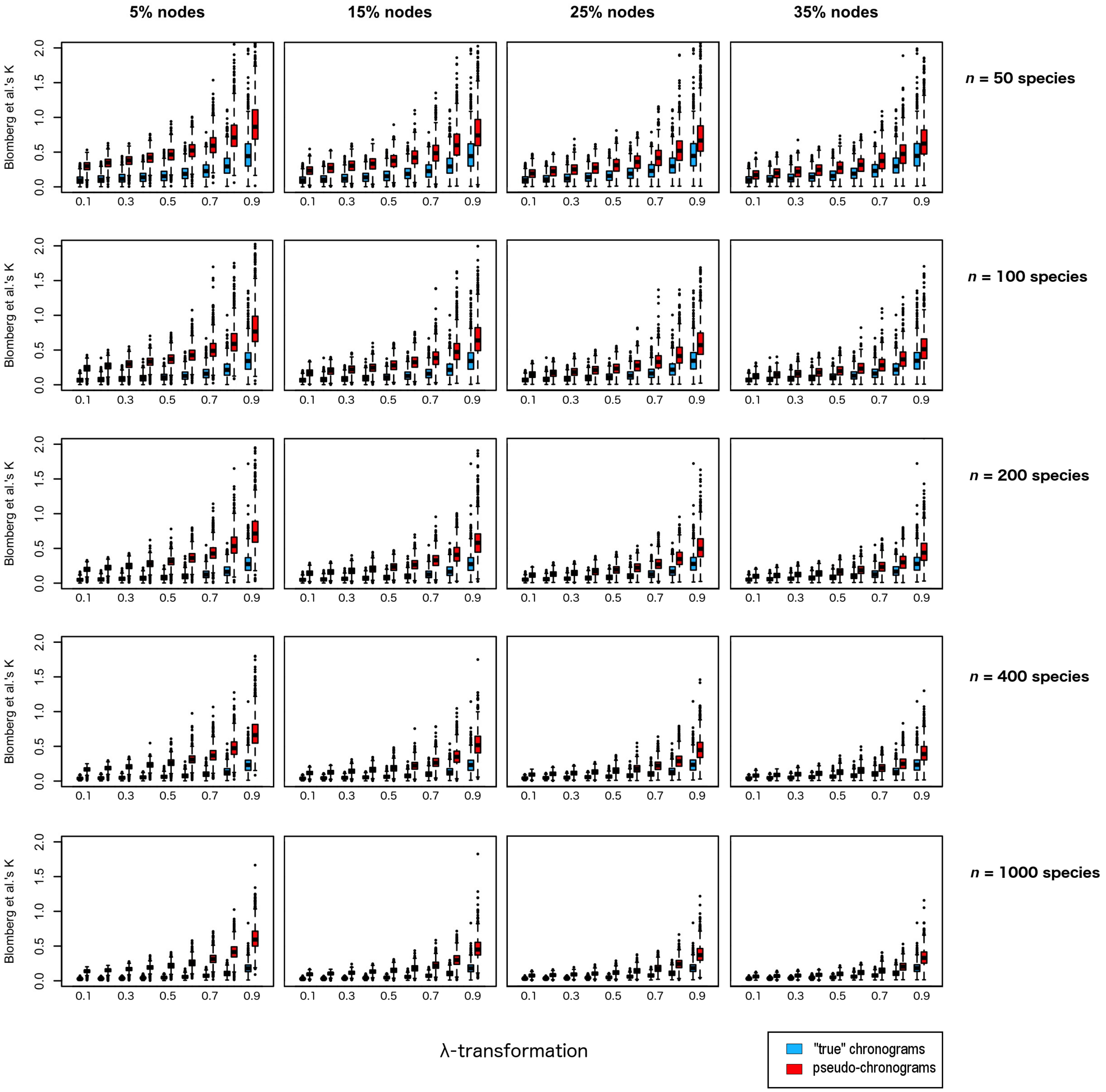

Figure S2. Values obtanied for Blomberg et al.'s *K* statistic under different scenarios of phylogenetic signal, sample size and branch-length information. The percentages above the top pannels represent the fraction of nodes that were fixed to calibrate the pseudo-chronograms (see main text).
