# Supplementary material for: Revisiting phylogenetic signal; strong or negligible impacts of polytomies and branch length information?
Source: BMC Evol Biol. 2017 Feb 15;17:53. doi: 10.1186/s12862-017-0898-y (PMC5312541; doi:10.1186/s12862-017-0898-y)
Supplement: Additional file 2: — Appendix 2. (values obtained for Blomberg et al.’s K and Pagels’s λ). (ZIP 4841 kb) [file 12862_2017_898_MOESM2_ESM.zip › Appendix 2 Figure S1.pdf]

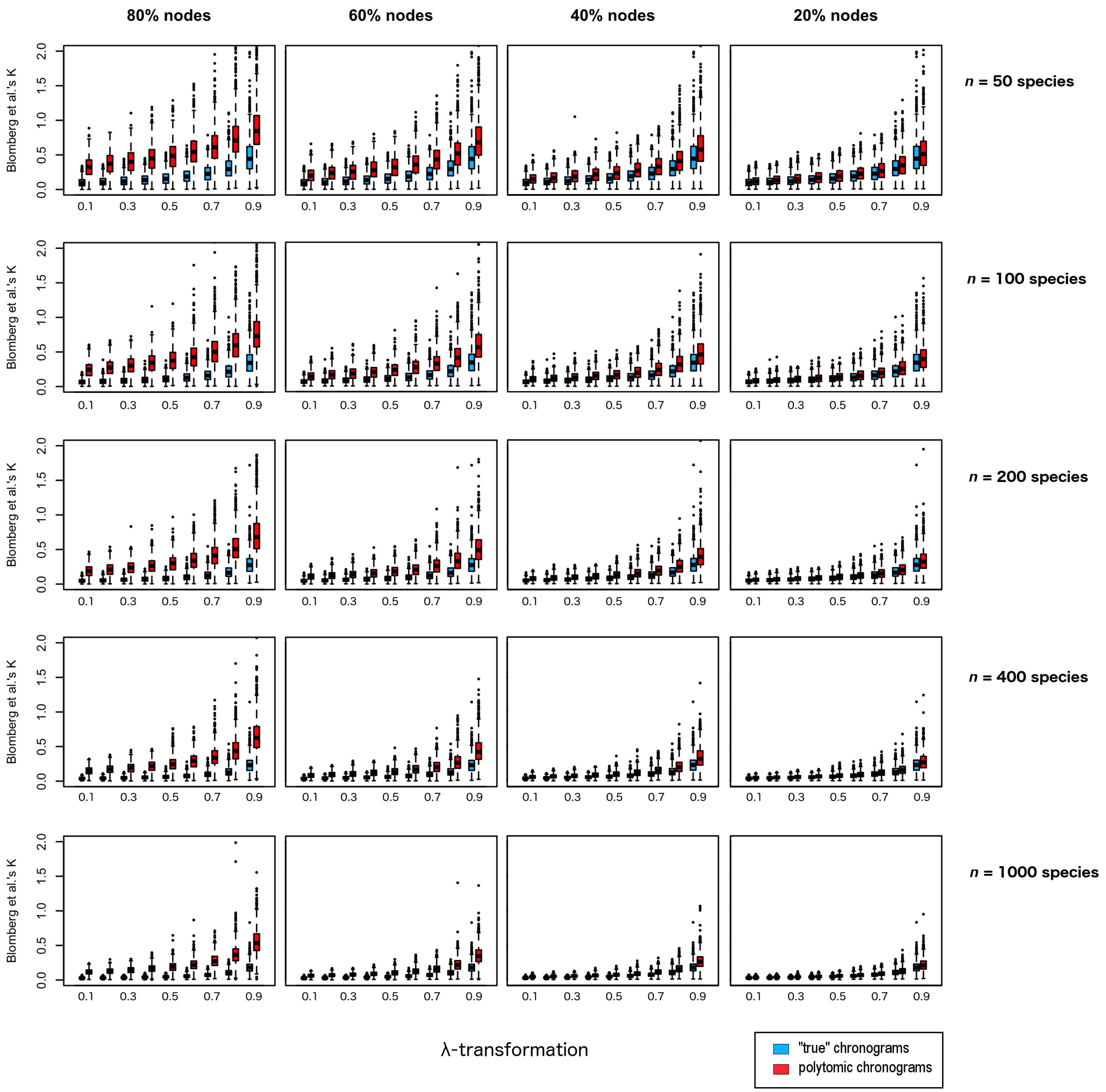

Figure S1. Values obtanied for Blomberg et al.'s *K* statistic under different scenarios of phylogenetic signal, sample size and phylogenetic resolution. The percentages above the top pannels represent the fraction of nodes placed above half of the height of the trees that were randomly collapsed to generate the polytomic chronograms (see text).
