# Supplementary material for: Revisiting phylogenetic signal; strong or negligible impacts of polytomies and branch length information?
Source: BMC Evol Biol. 2017 Feb 15;17:53. doi: 10.1186/s12862-017-0898-y (PMC5312541; doi:10.1186/s12862-017-0898-y)
Supplement: Additional file 3 — Appendix 3. (R code to simulate “true” chronograms and to generate polytomic chronograms and pseudo-chronograms). (PDF 62 kb) [file 12862_2017_898_MOESM3_ESM.pdf]

Appendix 3. R code to simulate “true” chronograms and to generate polytomic chronograms and pseudo-chronograms.

```
######################################
# R code to generate "true" chronograms  #
######################################

require(phytools)
N_Taxa=100

for(z in 1:1000){
pbtree(n=N_Taxa)->tree
write.tree(tree,paste("tree",z,sep=""))
print(paste("Tree",z,"saved",sep=""))
}


###########################################################################
#  R code to generate polytomic chronograms following the shallow-nodes strategy   #
###########################################################################

require(spacodiR)

Perc = 20 # Percentage of the nodes placed above half of the height of the given “true” chronogram to be collapsed

tree<-read.tree("User/.../") # load "true" chronogram in Newick format

Nodes1<- phy.nodetimes(tree,time.range=c(0.5,0))
Nodes1<- Nodes1[Nodes1!=0]
Nodes1<- as.integer(names(Nodes1))

if(length(Nodes1)==0){To_collaps1<-"no_procede"} else
{To_collaps1 <- round((length(Nodes1)*Perc)/100)}
if(To_collaps1==0) {To_collaps1<-1} else
{Sys.sleep(0.00000000000001)}

if(To_collaps1=="no_procede"){Sys.sleep(0.0000000000001)} else
{

for(q in 1:To_collaps1){
   Nodes1<-phy.nodetimes(tree,time.range=c(0.5,0))
         Nodes1 <- Nodes1[Nodes1!=0]
         Nodes1<-as.integer(names(Nodes1))

if (length(Nodes1)==0) {break} else

{if(length(Nodes1)==1) {
```

```
    Burt<-Nodes1[1]
    ll<-tree$edge.length[which(tree$edge[,2]==Burt)]
    tree$edge.length[which(tree$edge[,1]==Burt)]<-
    tree$edge.length[which(tree$edge[,1]==Burt)]+ll
    tree$edge.length[which(tree$edge[,2]==Burt)]<-0
    tree<-di2multi(tree)
  } else

{   Burt<-sample(Nodes1,1)
    ll<-tree$edge.length[which(tree$edge[,2]==Burt)]
    tree$edge.length[which(tree$edge[,1]==Burt)]<-
    tree$edge.length[which(tree$edge[,1]==Burt)]+ll
    tree$edge.length[which(tree$edge[,2]==Burt)]<-0
    tree<-di2multi(tree)

}}}}

write.tree(tree,"tree_Poly")


####################################################################
#   R code to generate polytomic chronograms following the all-nodes strategy   #
####################################################################

require(ape)

Species = 50 # number of tips in the “true” chronogram
Perc = 20 # Percentage of the nodes of the given “true” chronogram to be collapsed

tree<-read.tree("User/.../") # load "true" chronogram in Newick format

Total<- Species-2
To_collaps <-  round((Total*Perc)/100)

gg<-function(tree,n){
  for(i in 1:n){
    ii<-sample(2:tree$Nnode,1)+length(tree$tip.label)
    ll<-tree$edge.length[which(tree$edge[,2]==ii)]
    tree$edge.length[which(tree$edge[,1]==ii)]<-
      tree$edge.length[which(tree$edge[,1]==ii)]+ll
    tree$edge.length[which(tree$edge[,2]==ii)]<-0
    tree<-di2multi(tree)
  }
  tree
}

tree<-gg(tree,To_collaps)
write.tree(tree,"tree_Poly")
```

```
#####################################################################
#  R code to select nodes across across time-slices of the "true" chronograms   #
#####################################################################
```

Note: the following script generates the files to be used in Phylocom (BLADJ) in order to generate the pseudo-chronogram. The file "ages" contains the nodes selected to be fixed, and the file "phylo" contains the "true" chronogram to be used as input

```
require(spacodiR)
require(phytools)

Species=100 # number of tips in the “true” chronogram
Perc = 15   # Percentage of the nodes of the given “true” chronogram to be selected

tree<-read.tree("User/.../") # load "true" chronogram in Newick format

Total<- Species-1
To_fix <-  round((Total*Perc)/100)

Nodes1<-phy.nodetimes(tree,time.range=c(1,0.8))
Nodes1<-as.integer(names(Nodes1))

if(length(Nodes1)==0){To_fix1<-"no procede"} else
{To_fix1 <- round((To_fix)*length(Nodes1)/Total)}
if(To_fix1==0) {To_fix1<-1} else
{Sys.sleep(0.00000000000001)}

Nodes2<-phy.nodetimes(tree,time.range=c(0.8,0.6))
Nodes2<-as.integer(names(Nodes2))

if(length(Nodes2)==0){To_fix2<-"no procede"} else
{To_fix2 <- round((To_fix)*length(Nodes2)/Total)}
if(To_fix2==0) {To_fix2<-1} else
{Sys.sleep(0.00000000000001)}

Nodes3<-phy.nodetimes(tree,time.range=c(0.6,0.4))
Nodes3<-as.integer(names(Nodes3))

if(length(Nodes3)==0){To_fix3<-"no procede"} else
{To_fix3 <- round((To_fix)*length(Nodes3)/Total)}
if(To_fix3==0) {To_fix3<-1} else
{Sys.sleep(0.00000000000001)}

Nodes4<-phy.nodetimes(tree,time.range=c(0.4,0.2))
Nodes4<-as.integer(names(Nodes4))

if(length(Nodes4)==0){To_fix4<-"no procede"} else
{To_fix4 <- round((To_fix)*length(Nodes4)/Total)}
if(To_fix4==0) {To_fix4<-1} else
{Sys.sleep(0.00000000000001)}
```

```
Nodes5<-phy.nodetimes(tree,time.range=c(0.2,0))
Nodes5 <- Nodes5[Nodes5!=0]
Nodes5<-as.integer(names(Nodes5))

if(length(Nodes5)==0){To_fix5<-"no procede"} else
{To_fix5 <- round((To_fix)*length(Nodes5)/Total)}
if(To_fix5==0) {To_fix5<-1} else
{Sys.sleep(0.00000000000001)}

### Node picking ###

if(To_fix1=="no procede"){Slice1<-NA} else
{
if(length(Nodes1)==1 | To_fix1==1) {Slice1<-Nodes1[1]} else
{Slice1<-c(min(Nodes1),sample(Nodes1[-1],To_fix1))}}

if(To_fix2=="no procede"){Slice2<-NA} else
{
if(length(Nodes2)==1){Slice2<-Nodes2[1]} else
{Slice2<-sample(Nodes2,To_fix2)}}

if(To_fix3=="no procede"){Slice3<-NA} else
{
if(length(Nodes3)==1){Slice3<-Nodes3[1]} else
{Slice3<-sample(Nodes3,To_fix3)}}

if(To_fix4=="no procede"){Slice4<-NA} else
{
if(length(Nodes4)==1){Slice4<-Nodes4[1]} else
{Slice4<-sample(Nodes4,To_fix4)}}

if(To_fix5=="no procede"){Slice5<-NA} else
{
if(length(Nodes5)==1){Slice5<-Nodes5[1]} else
{Slice5<-sample(Nodes5,To_fix5)}}

###

Nodos_F <- c(Slice1,Slice2,Slice3,Slice4,Slice5)
Nodos_F <- Nodos_F[!is.na(Nodos_F)]

makeNodeLabel(tree, method = "number")->tree
paste("Node",Nodos_F-Species,sep="")->Nodos_bladj

DF_bladj<-matrix(nrow=length(Nodos_F),ncol=2)
DF_bladj<-as.data.frame(DF_bladj)

Nodos_bladj->DF_bladj[,1]
```

```
max(nodeHeights(tree))->tree_heigh

for(i in 1:length(Nodos_F)){

tree_heigh-nodeheight(tree, Nodos_F[i])->DF_bladj[i,2]

}

write.table(DF_bladj,quote=FALSE,row.names=FALSE,col.names=FALSE,"ages")
write.tree(tree,"phylo")
```
